# Molecular Characteristics of Carbapenem-Resistant *Enterobacter cloacae* in Ningxia Province, China

**DOI:** 10.3389/fmicb.2017.00094

**Published:** 2017-01-31

**Authors:** Zhiyun Shi, Huizheng Zhao, Gang Li, Wei Jia

**Affiliations:** ^1^Medical Experimental Center, General Hospital of Ningxia Medical UniversityYinchuan, China; ^2^Key Laboratory of Ningxia Clinical Pathogenic MicroorganismsYinchuan, China; ^3^Department of Laboratory, Institute of Clinical Medicine, Ningxia Medical UniversityYinchuan, China; ^4^Hematology and Oncology Center, Yanda Hospital of Hebei ProvinceLangfang, China

**Keywords:** *Enterobacteriaceae*, carbapenem resistance, antibiotic susceptibility, New Delhi metallo-beta-lactamase-1, genetic homologeneity

## Abstract

The emergence of carbapenem-resistant *Enterobacteriaceae* (CRE) has become a major public health concern worldwide and a new challenge in the treatment of infectious diseases. The molecular characteristics of *Enterobacter cloacae* in Ningxia China are unknown. In this study, we reported 10 carbapenem-resistant *E. cloacae* isolates from the General Hospital of Ningxia Medical University, the largest university hospital in Ningxia between January 2012 and December 2013. Bacteria isolates were identified by Vitek2 compact and the identity of non-duplicate *E. cloacae* isolates was further confirmed by PCR and sequencing. The drug susceptibility and phenotype identification of these isolates were analyzed by agar dilution method, modified Hodge test (MHT), and EDTA synergy test. Beta-lactamase (bla) genes *bla*_NDM−1_ was found in 8 out of 10 isolates. Most isolates harbored multiple resistance genes including *bla*_ESBL_, *bla*_AmpC_, quinolones, aminoglycosides, and disinfectant resistance genes. Pulsed field gel electrophoresis (PFGE) showed that these *E. cloacae* isolates were grouped into 6 clusters based on a cutoff of 80% genetic similarity. In conjugative assay, 9 out of 10 isolates transferred carbapenem-resistant genes to *Escherichia coli*. Our study has revealed that NDM-1-producing isolates are the most prevalent carbapenem-resistant *E. cloacae* in Ningxia. These isolates also carry several other carbapenem-resistant genes and can transfer these genes to other bacteria through conjugation. These findings highlight an urgent need to monitor these isolates to prevent their further spread in this region.

## Introduction

The *Enterobacteriaceae* are a large family of Gram-negative bacteria that may cause several infectious diseases such as respiratory tract infection, intra-abdominal infection, urinary tract infection, etc. (Wang J. T. et al., 2015). Carbapenem has been the main treatment for severe infections associated with *Enterobacteriaceae* (Kaniga et al., 2010). Nevertheless, carbapenem-resistant *Enterobacteriaceae* (CRE) has gradually emerged and becomes one of the serious public health concerns worldwide (Alam et al., 2014; Ling et al., 2015; Xu et al., 2015; Iredell et al., 2016). According to the 2012 CHINET report on CRE in China, the domestic incidence of CRE has increased 2.2-fold between 2010 and 2012 (Hu et al., 2012). Carbapenem resistance in *Enterobacteriaceae* is mainly mediated by the production of carbapenemases, a form of β-lactamase, that cleave the β-lactam ring, an essential component of β-lactam antibiotics such as cephalosporins and carbapenems (Queenan and Bush, 2007).

*Enterobacter cloacae*, a species of the *Enterobacteriaceae* family, is an important nosocomial pathogen that may cause infections in wound, urinary tract, lower respiratory tract, skin and soft tissues. While CRE infections are primarily caused by *Klebsiella pneumoniae* and *Escherichia coli* (Goodman et al., 2016), carbapenem-resistant *E. cloacae* has only occasionally been reported (Bennett et al., 2009; Heller et al., 2012). In this study, we reported 10 carbapenem-resistant *E. cloacae* isolated in the largest university hospital in Ningxia province, China and investigated their molecular characteristics.

## Materials and methods

### Reagents

Imipenem, meropenem, ceftazidime, and tigecycline was purchased from Pfizer. MH agar was purchased from OXOID. Primers were synthesized by Sangon Biotech Shanghai Co. Ltd. Premix Taq was purchased from Takara Biotech Dalian Co. Ltd. Xba I restriction enzymes were purchased from Thermo.

### Sample collection, bacterial identification, and reference strains

Bacterial isolates were collected from the General Hospital of Ningxia Medical University, a large-sized hospital in Ningxia province, China, between January 2012 and December 2013. Species identification and initial drug susceptibility were analyzed by Vitek2 compact (bioMerieux, France). *E. coli* ATCC25922, *Klebsiella pneumonia* ATCC BAA-1705, and *K. pneumonia* ATCC BAA-1706 were used as quality control strains.

### Identification of *E. cloacae* by PCR and sequencing

The identity of all *E. cloacae* isolates was further confirmed by PCR and sequencing. Briefly, the isolates were cultured by standard methods. To isolate chromosomal DNA, one freshly grown colony was scraped into an Eppendorf tube and resuspended in 200 μL of sterile water. The bacterial suspension was boiled at 100°C for 10 min to release the genomic DNA. The DNA was then used as the template to amplify the 16S rRNA gene segment (1465 bp) by PCR. The sequences of primers: 27F: AGA GTT TGA TCM TGG CTC AG, and 1492R: TAC GGY TAC CTT GTT ACG ACT T. The PCR programs were as follows: denaturation at 94°C 5 min, followed by 30 cycles of 94°C 30 s, 55°C 40 s, and 72°C 1 min, and extension at 72°C 5 min. The PCR products were sequenced by BGI Genomics (Beijing, China).

### Drug susceptibility test and phenotype identification

The antibiotic susceptibilities of the *E. cloacae* isolates were tested by measuring the minimal inhibitory concentration (MIC) for imipenem, meropenem and tigecycline using the agar dilution method. Imipenem and meropenem results were interpreted according to the Clinical Laboratory Standards Institute (CLSI) guidelines (2013). Freshly prepared tigecycline (<12 h old) was used, and results were interpreted based on the FDA criteria (R:MIC ≥ 8, I:MIC = 4, S:MIC ≤ 2). All *E. cloacae* isolates were screened for carbapenemase production by the modified Hodge test (MHT) and EDTA synergy test according to the CLSI guidelines. In EDTA synergy test, 0.5M units bacterial sample was spread on MH agar plate. Two meropenem disks were placed on the plate with a distance of 15–20 mm. Aliquots of EDTA (10 μL of 0.5 mol/L solution) was added onto one of the disks, and the plate was incubated at 35°C for 18–24 h. A positive result was scored when the diameter of the inhibition zone around EDTA disk was 5 mm larger than that around the other disk. Both MHT and EDTA synergy test were performed in triplicates.

### Detection of resistance genes

The DNA was extracted from each carbapenem-resistant *E. cloacae* isolate. Resistance genes were detected by PCR, including extended-spectrum β-lactamases (ESBL: *bla*_TEM_, *bla*_SHV_, *bla*_CTX_, *bla*_VEB_, *bla*_PER,_ *and bla*_SFO_), AmpC enzyme (*bla*_DHA_, *bla*_EBC_, *bla*_ACC_, *bla*_CIT,_ *and bla*_MOX_), carbapenemase (*bla*_KPC_, *bla*_NDM−1_, *bla*_SME_, *bla*_IMP_, *bla*_VIM_, *bla*_OXA−23,_ *and bla*_OXA−48_), class 1 and 2 integron, and other resistant genes (qnrA, qnrB, qnrS, acc-Ib, qacΔE, and sul). The reaction system (25 μL) was prepared: Premix Taq 12.5 μL, template 2 μL, each primer 0.2 μL (~10 μM), and sterile water 10.1 μL. Reaction program was as follows: 94°C denaturation for 5 min, followed by 30 cycles of 94°C denaturation for 45 s, 52–58°C annealing for 45 s, 72°C extension for 1 min, 72°C final extension for 10 min. Positive amplification products were sequenced and the sequencing results were compared by BLAST.

### Genetic homologeity analysis

All carbapenem-resistant *E. cloacae* were analyzed by pulsed field gel electrophoresis (PFGE) to determine their genetic homologeity. The bacterial DNA embedded in small plastic pieces was treated with proteinase K and cleaved by the restriction enzyme XbaI at 37°C for 12–14 h. The XbaI-digested DNA was analyzed by electrophoresis for 21 h at 6 V/cm, with a pulse angle of 120° and pulse times from 2.16 to 63.8 s. PFGE was performed in triplicates. Comparison of the PFGE patterns was performed with Bionumerics software based on the Tenover's criteria (Tenover et al., 1995). Isolates were allocated into genetic similarity clusters using a cut-off value of 80%.

### Plasmid conjugation test

The conjugation test was performed by the broth mating method using carbapenem-resistant *E. cloacae* as the donor strain and rifampin-resistant *E. coli EC600* as the recipient strain. The donor and recipient strain were inoculated respectively in 5 mL LB, and was incubated in a thermostatic shaker at 37°C for 18–24 h. The donor strain (60 μL) and recipient strain (90 μL) were inoculated into 20 ml of LB medium and incubated 37°C for 18–24 h without any shaking. Transconjugants were selected on MH agar plates containing 30 μg/mL ceftazidime + 400 μg/mL rifampicin and plates with 1 μg/mL imipenem + 400 μg/mL rifampicin. The donor and recipient strains were used as negative controls. The transconjugants strains were identified by PCR and sequencing. The MIC for imipenem, meropenem, and tigecycline was measured by the agar dilution method. The conjugation test was performed in triplicates

## Results

### Phenotype and genotype of carbapenem-resistant *E. cloacae* isolates

A total of 18 non-duplicate CRE isolates were identified from 2012 to 2013, 10 of which were *E. cloacae*. Eight and nine positive *E. cloacae* isolates were detected in the MHT and EDTA double disk synergy test, respectively. PCR amplification showed that these *E. cloacae* isolates carried multiple resistant genes. Integrons and insertion sequence common regions (ISCR) were present in 8 and 9 out of 10 *E. cloacae* isolates, respectively. As shown in Table 1, the carbapenem-resistant gene was primarily New Delhi metallo-beta-lactamase-1 (*bla*_NDM−1_, 8/10), followed by *K. pneumoniae* carbapenemase (*bla*_KPC_, 6/10). ESBL, AmpC, quinolones, aminoglycosides and disinfectant resistance gene was found in 7, 8, 8, 4, and 8 out of 10 isolates, respectively.

**Table 1:** **Summary of resistant genes detected in the carbapenem-resistant ***E. cloacae*** isolates**.

**Isolate ID**	**MHT/EDTA double-disk synergy test**	**Associated resistance determinants**	
		**β-lactamase gene**	**Others**
3	+/+	*bla*_TEM_, *bla*_KPC_, *bla*_NDM−1_, *bla*_IMP_, *bla*_VIM_	qacΔE, IntI, ISCR
9	+/+	*bla*_CTX−1_, *bla*_EBC_, *bla*_KPC_, *bla*_NDM−1_, *bla*_VIM_	acc-Ib, qacΔE, sul, IntI, ISCR
11	+/+	*bla*_ACC_, *bla*_EBC_, *bla*_KPC_, *bla*_NDM−1_	qnrA, qacΔE, IntI, ISCR
18	+/+	*bla*_CTX−1_, *bla*_EBC_, *bla*_KPC_, *bla*_NDM−1_	qnrA, qacΔE, sul, IntI, ISCR
40	±	*bla*_TEM_, *bla*_SHV_, *bla*_ACC_, *bla*_EBC_, *bla*_NDM−1_, *bla*_VIM_	acc-Ib, qacΔE, sul, IntI, ISCR
42	+/+	*bla*_TEM_, *bla*_KPC_, *bla*_NDM−1_, *bla*_VIM_	qnrS, acc-Ib, qacΔE, sul, IntI, ISCR
55	+/+	*bla*_IMP_	–
56	+/+	*bla*_TEM_, *bla*_DHA_, *bla*_EBC_, *bla*_NDM−1_	acc-Ib, qacΔE, sul, IntI, ISCR
02	−/−	*bla*_ACC_, *bla*_EBC_	ISCR
03	+/+	*bla*_CTX−1_, *bla*_DHA_, *bla*_EBC_, *bla*_KPC_, *bla*_NDM−1_, *bla*_VIM_	qnrA, qacΔE, sul, IntI, ISCR

### Molecular epidemiology

**Figure 1:**
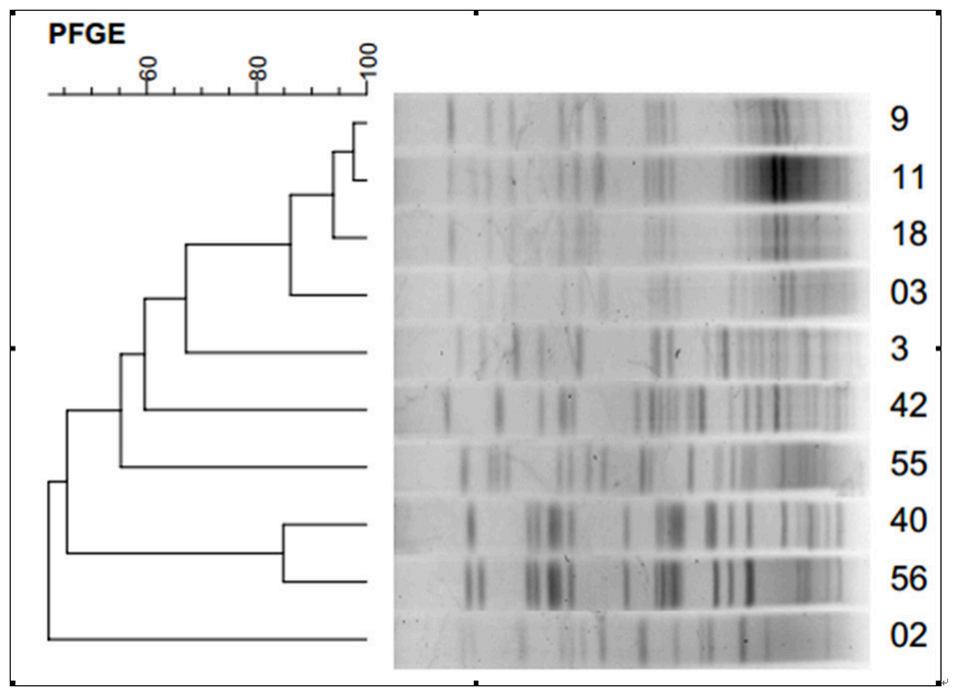
**Dendrogram showing pulsed-field gel electrophoresis (PFGE) analysis of the 10 carbapenem-resistant ***E. cloacae*** isolates**.

Based on a cutoff of 80% genetic similarity, these isolates were grouped into A (isolate No: 9, 11, 18, 03), B (isolate No: 33), C (isolate No: 42), D (isolate No: 55), E (isolate No: 40, 56), and F (isolate No: 02) clusters (Figure 1).

### Plasmid conjugation test

The plasmid transconjugant test was performed for each carbapenem-resistant *E. cloacae* isolate. The transconjugants were analyzed by PCR and sequencing. Results showed that a total of 9 transconjugants were *E. coli*, indicating the successful transfer of resistant plasmid from *E. cloacae* isolate to *E. coli*. The MIC of the 9 transconjugants for imipenem, meropenem and tigecycline was measured by the agar dilution method (Table 2).

**Table 2:** **Minimum inhibitory concentrations (MIC) for imipenem, meropenem and tigecycline of the carbapenem-resistant ***E. cloacae*** isolates and their transconjugants**.

**Isolate**	**S/F**	**IPM**	**TC-IPM**	**MEM**	**TC-MEM**	**Tigecycline**	**TC-Tigecycline**
3	S	4	4	8	8	1	1
9	S	64	4	32	1	1	≤0.125
11	S	≥128	4	≥128	4	0.5	1
18	S	64	2	32	8	8	1
40	S	64	32	32	32	8	8
42	S	8	4	8	16	8	2
55	S	4	4	4	4	32	8
56	S	32	32	32	16	8	4
02	F	–	–	–	–	–	–
03	S	16	4	8	4	0.5	1
EC600	–	0.5	–	≤0.125	–	≤0.125	–

*S/F, success/failure; IMP, impipenem; MEM, meropenem; TC, transconjugant strain; and EC600, recipient strain*.

## Discussion

Carbapenem-resistant *E. cloacae* can markedly prolong the hospital length of stay, increase the medical cost, and most importantly cause high mortality rate. With the wide spread of CRE and associated resistant genes around the word (Castanheira et al., 2013; Poirel et al., 2014; Zweigner et al., 2014; Jakobsen et al., 2015), it has become urgent to investigate the epidemiology and molecular characteristics of carbapenem-resistant *E. cloacae*, which may provide a solid basis for an effective control of carbapenem-resistant *E. cloacae*.

In this study, 55.6% of the carbapenem-resistant *E. cloacae* isolated in our hospital can produce carbapenemase, which is substantially different from the CHINET report (Ling et al., 2015). The finding has reflected the distinct distribution of the bacteria in this region, which might be related to the antibiotic-prescribing habits of local doctors. After NDM-1 was first reported by Kumarasamy in 2010 (Wang et al., 2011), NDM-1-producing *E. cloacae* have been identified throughout the world (Gupta et al., 2011; Ho et al., 2012). In China, NDM-1 was initially detected in Ningxia province and has been later reported in other regions (Ho et al., 2012; Huang et al., 2015; Zhang et al., 2015). In this study, the carbapenem-resistant gene is primarily *bla*_NDM−1_, and other carbapenemase genes are also detected including *bla*_KPC_, imipenem-hydrolizing-lactamase gene (*bla*_IMP_), and Verona imipenemase gene (*bla*_VIM_). Inconsistent with our results, *bla*_KPC−2_ and *bla*_VIM−1_ were identified as the primary carbapenem-resistant genes of CRE isolates (Levy Hara et al., 2013; Li et al., 2014; Villa et al., 2014). More importantly, in our study, non-carbapenemase-producing resistant genes such as β-lactamase, quinolones, aminoglycosides, etc. were detected. The gene of integration, ISCR and other genetic elements were also identified in these isolates, suggesting the possibility of exogenous gene transfer. Two of the isolates even carry six β-lactamases and some other resistance genes. Our results have revealed a diversity of resistance genes in our isolates, indicating that these isolates might have achieved resistance through different mechanisms.

Ningxia province, a relatively less developed region in the northwest of China, has a much lower risk of bacterial transmission compared with other regions (Hu et al., 2014; Ling et al., 2015). In this study, the patients carrying the carbapenem-resistant *E. cloacae* isolates had no prior travel or living in any epidemic areas, indicating that these isolates might have acquired the carbapenem resistance gene through a unusual way. Moreover, *Acinetobacter baumannii* had been previously isolated from 6 of these carrying patients, suggesting the probable transmission of resistant genes between different bacterial species. Such cross-species gene transmissions have been previously detected (Bogaerts et al., 2013; Wang X. et al., 2015).

MHT is recommended for the carbapenemas phenotype identification test for by CLSI. Studies have shown that the sensitivity of MHT for some strains is much higher compared with others containing IMP, VIM, and NDM (a sensitivity of 50%; Girlich et al., 2012; Ramana et al., 2013). In this study, the positive rate of MHT is 8/10, indicating the occurrence of false negative, which might be associated with the production of IMP, VIM, and NDM in some isolates. EDTA can inactivate the type B carbapenemas by chelating Zn ions (Miriagou et al., 2013), resulting in the expansion of the inhibition zone in EDTA synergy test. In this study, the positive rate of EDTA synergy test is 9/10, and most of the positive strains produce the type B carbapenemas. Although, Azimi et al. have suggested that false negative results are associated with the test method instead of the type of carbapenemases (Azimi et al., 2016), Wang et al. have reported a false positive rate of 3.3% in MHT due to the low ertapenem hydrolysis capacity of ESBL, especially CTX-M (Wang et al., 2011). Therefore, a combination examination of carbapenemas phenotype by MHT and EDTA synergy test can improve the sensitivity and specificity of carbapenemas gene detection. Moreover, PCR method remains the gold standard for carbapenemas phenotype identification (Rastegar Lari et al., 2013). Consistently, the positive rate of PCR in our study is 10/10.

In this study, based on a cutoff of 80% genetic similarity, PFGE analysis revealed a high degree of genetic homologeity among 5 isolates, suggesting that small-scale epidemic spread might have occurred. Further transconjugation assay has detected a positive rate of 9/10, which is markedly higher than that in a previous study (Fu et al., 2012). The finding suggests that our carbapenemase-producing *E. cloacae* can transfer the resistant genes to susceptible bacterium, leading to horizontal transfer of resistant genes, and thus an infection outbreak of the pathogens. Furthermore, our study also showed that the MIC of most transconjugates for imipenem, meropenem, and tigecycline was lower compared with their donor bacteria, indicating that the bacterial resistance is dependent on several factors. The mechanism of clinical transmission among different bacteria needs further investigation.

In summary, our study demonstrated that NDM-1-producing isolates were the most prevalent carbapenem-resistant *E. cloacae* in Ningxia province, China. These isolates can transfer the resistant gene to other bacteria through conjugation. To our best knowledge, this is the first report on the prevalence and molecular characteristics of carbapenem-resistant *E. cloacae* in, China. Early detection and surveillance of these *E. cloacae* isolates are urgently needed to prevent their further spread.

## Author contributions

WJ designed and supervise the study. ZS performed the experiments, analyzed the data, and wrote the paper. HZ helped with experiments. GL helped with the writing of the paper.

### Conflict of interest statement

The authors declare that the research was conducted in the absence of any commercial or financial relationships that could be construed as a potential conflict of interest.
